# Studies of the Influence of Beam Profile and Cooling Conditions on the Laser Deposition of a Directionally-Solidified Superalloy

**DOI:** 10.3390/ma11020240

**Published:** 2018-02-04

**Authors:** Shuo Yang, Dong Du, Baohua Chang

**Affiliations:** State Key Laboratory of Tribology, Department of Mechanical Engineering, Tsinghua University, Beijing 100084, China; yangs10@163.com (S.Y.); dudong@tsinghua.edu.cn (D.D.)

**Keywords:** laser metal deposition, directionally solidified superalloys, beam profiles, cooling conditions, microstructure

## Abstract

In the laser deposition of single crystal and directionally-solidified superalloys, it is desired to form laser deposits with high volume fractions of columnar grains by suppressing the columnar-to-equiaxed transition efficiently. In this paper, the influence of beam profile (circular and square shapes) and cooling conditions (natural cooling and forced cooling) on the geometric morphology and microstructure of deposits were experimentally studied in the laser deposition of a directionally-solidified superalloy, IC10, and the mechanisms of influence were revealed through a numerical simulation of the thermal processes during laser deposition. The results show that wider and thinner deposits were obtained with the square laser beam than those with the circular laser beam, regardless of whether natural or forced cooling conditions was used. The heights and contact angles of deposits were notably increased due to the reduced substrate temperatures by the application of forced cooling for both laser beam profiles. Under natural cooling conditions, columnar grains formed epitaxially at both the center and the edges of the deposits with the square laser beam, but only at the center of the deposits with the circular laser beam; under forced cooling conditions, columnar grains formed at both the center and the edges of deposits regardless of the laser beam profile. The high ratios of thermal gradient and solidification velocity in the height direction of the deposits were favorable to forming deposits with higher volume fractions of columnar grains.

## 1. Introduction

Single crystal (SX) and directionally-solidified (DS) superalloys are increasingly being used to manufacture critical hot components (e.g., turbine blades) in aeroengines and gas turbines. Defects like cracks and wear are inevitable for such superalloy components due to the high temperature and high pressure service environments [1,2]. Because of the very high cost involved in replacing these damaged components, it is of great economic significance to repair them so as to prolong their service life. During the repairing of components made of single crystal or directionally-solidified superalloys, epitaxial columnar grains with the same orientation as that of the base metal are preferable to the equiaxed grains because of their better performance [3]. As an advanced additive manufacturing technology, laser metal deposition has been considered as an ideal process to repair such superalloy components, because the concentrated heat input from a laser beam can form a high temperature gradient, which is in favor of the oriented growth of grains in solidifying deposits.

So far, research has already been carried out to study the columnar grain growth and the columnar-to-equiaxed transition (CET) in the laser metal deposition of SX and DS superalloys. Based on the first analytical model to describe the CET in solidifying processes [4], Gaumann et al. [5,6,7] studied the rapid solidification processes and presented a theoretical model describing the nucleation and growth processes of grains ahead of the solid–liquid interface in solidification. Results showed that the thermal gradient (G) and the solidification velocity (V) were critical in determining the resulting solidification microstructures in the laser metal deposition of a SX superalloy CMSX-4. Liu et al. [8] derived a simple relationship between the G/V ratio and process parameters (laser power, traveling speed, etc.), which was then used to control the grain growth in order to obtain oriented epitaxial columnar grains in the laser deposition of a SX Ni_3_Al-based alloy IC221W. Basak et al. [9,10] studied the qualities of single-pass deposits of SX CMSX-4 and Rene N5 fabricated by scanning laser epitaxy (SLE). Crack-free deposits with columnar heights larger than 500μm were obtained through computational modeling, experimental process development, and process parameter optimization. Liu et al. [11,12] systematically studied the effects of the process parameters (laser power, traveling speed, and powder feeding rate) on the crystal growth and microstructure, and developed a three- dimensional (3D) numerical model to simulate the crystal growth in multi-layer and multi-track laser deposition of a SX superalloy. Results showed that the overlapping ratio was very important to ensure the continuity and consistency of the epitaxial growth of columnar grains. Do et al. [13] studied the effect of the overlapping ratio of neighboring tracks on the growth of columnar dendrites in the laser deposition of a DS superalloy DZ125L, and showed that the epitaxial columnar dendrites could grow continuously between deposition tracks when the overlapping ratio was big enough, which however would result in uneven deposit layers. Although certain progress has been made in the laser metal deposition of SX and DS superalloys, the continuous growth of columnar dendrites with consistent orientation is still a big challenge. This is because the grains at the edges of a laser deposition track generally have random orientations and are not parallel to the crystal orientation of the base metal, which make it impossible for the columnar grains to grow continuously in the overlapping zone between two neighboring deposition tracks [13]. 

Obviously, for given substrates and powders, the grain growth in the laser deposits depends mainly on the temperature distribution and thermal history during laser deposition, which are determined by the heat input and cooling conditions during laser deposition [4,7,14]. Laser process parameters (laser power, traveling speed, etc.) directly affect the heat input, and further affect the thermal gradient and the solidification velocity at the front of the solid–liquid interface, which control the growth of microstructure. However, it is still difficult to obtain satisfying directional columnar grains only by optimizing the laser process parameters. Some researchers have used other methods to vary the thermal gradient and the solidification velocity to enlarge the laser process window for the growth of consistently directional columnar grains. Wang et al. [15] found that changing the substrate orientation by rotating the SX substrate for a proper angle was beneficial for avoiding CET and could widen the laser processing window during laser SX repair processes. Shang et al. [16] found that the homogeneity of the microstructure and the distribution of hard phases in the deposition layer of the NiCrBSi alloy could be controlled by varying the laser beam profiles (shapes of laser beam spots, and the distributions of laser energy within the beam spots). Goffin et al. [17] controlled the beam profile by holographic optical elements (HOEs) for wire-fed laser cladding of 316 stainless steel and showed that superior wetting behavior and a less dilution cladding layer were obtained by a more even beam profile. Chen et al. [18] changed the cooling conditions by applying cooling water to the substrates during the laser additive manufacturing of Inconel 718, and found that the consistency of the crystal orientation was improved and the susceptibility to liquation cracking was decreased by increasing the base cooling effect. 

So far, there is no research reported on the influence of beam profile and cooling conditions on the laser deposition of single crystal and directionally-solidified superalloys. In this paper, the influence was studied of two laser beam profiles (with different shapes and energy distributions) and two cooling conditions (natural cooling versus forced cooling) on the crystal growth in the laser deposition on a DS superalloy IC10. The results obtained are helpful in obtaining a high fraction of directional growth columnar grains by suppressing the CET in repairing components of SX or DS superalloys by laser metal deposition. 

## 2. Research Approaches

### 2.1. Experimental Research

The substrate material used in the present study is a DS Ni-based superalloy IC10, which has superior mechanical properties at temperatures as high as 1100 °C [19]. Table 1 lists the chemical composition of IC10, which was captured by a spectrum analyzer ICP6300. The DS IC10 alloy casts were cut through planes perpendicular to the solidification direction into sheet specimens with dimensions of 70 mm × 24 mm × 4 mm by wire cutting, of which the surfaces were then polished with sandpaper and cleaned with acetone. 

**Figure 1:**
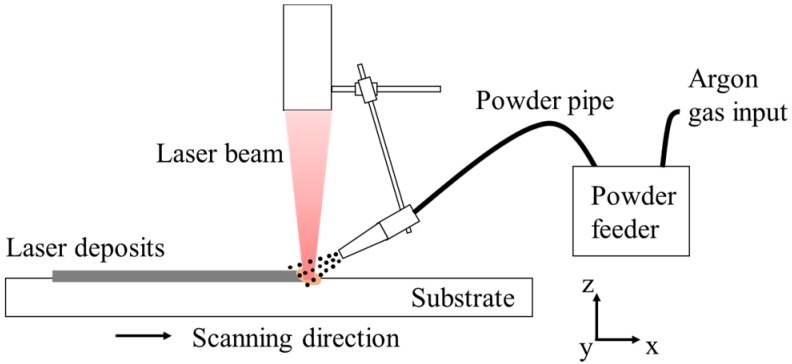
Schematic diagram of the laser deposition process with lateral powder feeding.

An IPG YLS-2000W fiber laser and DPSF-2 powder feeder were used in the laser deposition trials, as shown in Figure 1. The alloy powders, with diameters ranging from 50 μm to 150 μm and a chemical composition the same as that of the substrate, were fed through a lateral nozzle. The process parameters used were chosen based on previous research [20], i.e., laser power of 600 W, scanning speed of 3.5 mm/s, alloy powder feeding rate of 9.0 g/min, and shielding gas (argon) flow rate of 7.0 L/min. With these parameters determined, four sets of experiments, as listed in Table 2, were carried out with two different laser profiles and two different cooling conditions. 

**Figure 2:**
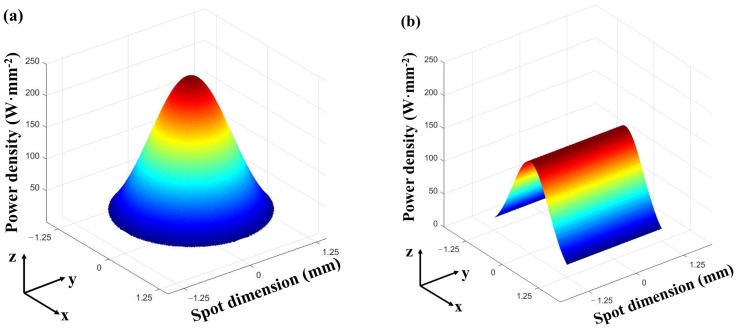
Power density distributions of the two laser beam profiles used when laser power is 600 W. (**a**) Circular beam spot of 2.5 mm diameter; (**b**) square beam spot of 2.5 × 2.5 mm.

Figure 2 shows the two profiles of the laser beam used in this study. Figure 2a is the circular laser beam spot of 2.5 mm diameter with Gaussian power density distribution, and Figure 2b is the square laser beam spot of 2.5 mm side length with a uniform power density in one (y) direction and a Gaussian power density distribution in the other (x) direction. 

**Figure 3:**
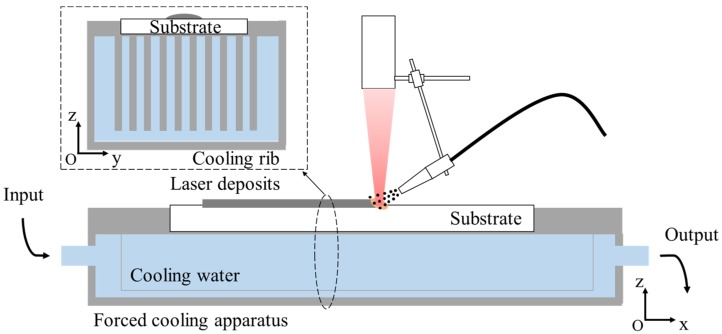
The forced cooling condition used in laser deposition.

Figure 3 schematically shows how the forced cooling conditions were realized by flowing water beneath the specimen being laser deposited. The customized water cooling fixture was made from copper with high thermal conductivity, and the distance between the bottom surface of the specimen and the flowing water was 3.0 mm. For natural cooling, the specimens were fixed directly on the worktable and no special cooling measure was used. 

A single-track deposit was made on each specimen, and three specimens were made for each of the four sets of process parameters. Metallographic samples were prepared by first cutting through the cross sections of the deposits by wire-cutting, which were then ground, polished, and chemically etched with a solution of 4 g CuSO_4_ + 20 mL HCl + 20 mL H_2_O. The geometric morphology and metallurgical microstructure of the deposits made with different sets of process parameters were observed by OLYMPUS DP2 optical microscopy (OM). The averages were taken over the measurements on the geometric profiles of three deposits for each parameter set to avoid influences from random factors.

### 2.2. Numerical Analysis

**Figure 4:**
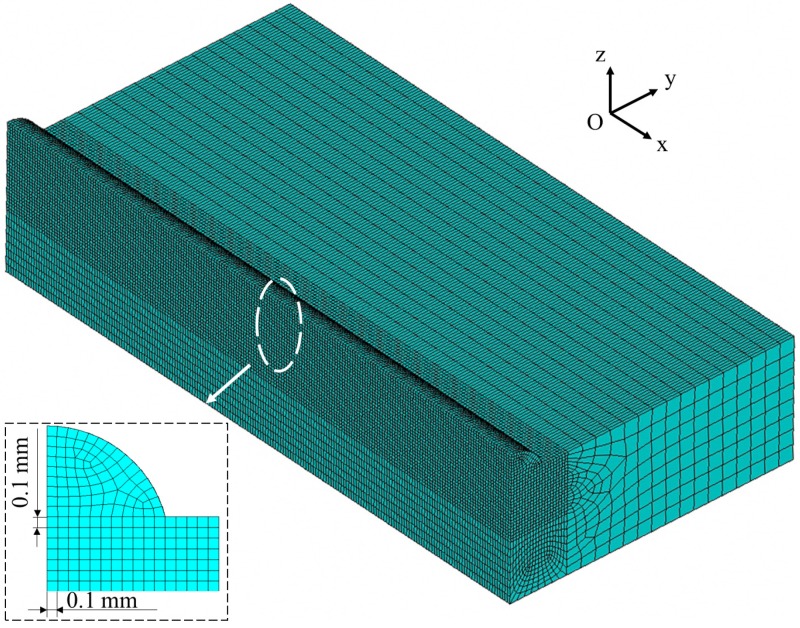
Finite element mesh used in the numerical simulation.

To understand the mechanisms of the phenomena revealed by the laser deposition experiments, a numerical model was developed to analyze the thermal processes (temporal and spatial distribution of temperatures) during laser deposition with the finite element analysis code ANSYS. As the specimens and heat sources were both symmetrical about the central plane of a specimen, only one half of the specimen was included in the finite element mesh to improve the computational efficiency, as shown in Figure 4. The dimensions of the substrates in the models were 70 mm × 12 mm × 4 mm and the dimensions of the deposits were in agreement with those obtained in experiments and were different for the four deposition conditions. The eight-node hexahedral element Solid70 was used to mesh the computational region. Finer elements were used in the deposits and heat affected zones (HAZ) with the minimum dimensions of 0.1 mm × 0.1 mm × 0.1 mm, while coarser elements were used in the regions away from deposits and HAZ. A convergence study was carried out to guarantee that the finite element mesh was fine enough, in which both maximum temperatures and the dimensions of the molten pools (isotherms) were compared for different mesh sizes. 

The heat flux (power density) for the circular laser beam spot of 2.5mm diameter with a Gaussian distribution, as shown in Figure 2a, was determined by the following equation:

(1) $$P\left(r\right)=\frac{2P_{c}}{\pi R^{2}}exp\left(-\frac{2r^{2}}{R^{2}}\right)$$

in which, *P*(*r*) was the heat flux within the circular beam spot, *P_c_* was the input power of laser beam, *R* was the spot radius, *r* was the distance to the center of the beam spot.

The heat flux for the square beam spot of 2.5 mm side length shown in Figure 2b was described as follows:

(2) $$P\left(x,y\right)=\frac{P_{s}}{l^{2}\sqrt{2\pi }}exp\left(-\frac{2x^{2}}{l^{2}}\right)$$

where *P*(*x*, *y*) was the heat flux within the square beam spot, *P_s_* was the input power of laser beam, *l* was half of the side length of the square spot. The heat flux had a Gaussian distribution in the scanning (x) direction and a uniform distribution in the direction of the deposit width (y). 

A natural convection boundary was applied to the bottom of the specimen to simulate the natural cooling condition with a convection heat transfer coefficient of 20 W/m^2^K, while the forced cooling conditions by flowing water were modeled by specifying the temperature to be 20 °C on the bottom surface of the specimen.

The process parameters used in the simulation were the same as those used in the experiments, i.e., laser power of 600 W and a scanning speed of 3.5 mm/s. The thermo-physical parameters of IC10 used in the numerical modeling are shown in Table 3. 

A laser deposition process of 4 s was numerically modeled for each set of parameters. A quasi-steady state was reached within the deposition time simulated, which meant that the temperature fields no longer changed with the increase of the deposition time. In the simulation, the laser deposition process was divided into a series of small time steps. The initial time step used was the ratio of the minimum grid spacing and scanning speed (0.0287 s), and size of the time sub-step was automatically adjusted by the ANSYS program based on the criteria set in the program for the solution accuracy and computational efficiency. 

The ‘birth and death’ capability of the element was used to simulate the additive process of the powders. That is to say, the elements representing deposits were in the ‘death’ state before the laser heat source was applied, and were activated to the ‘birth’ state when the laser heat flux was applied. 

## 3. Experimental Results

### 3.1. Geometric Morphology of Deposits

**Figure 5:**
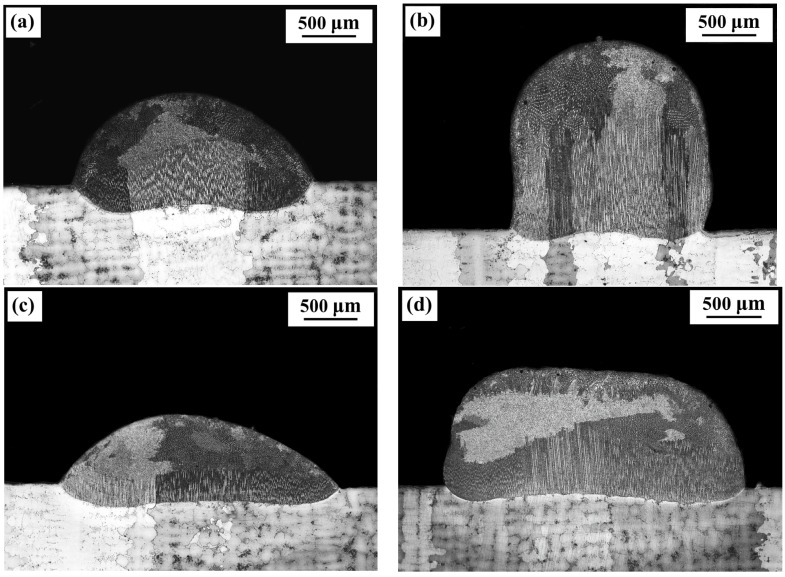
Geometric morphology of the cross-sections of deposits made with four conditions (observed with an OM). (**a**) Circular beam and natural cooling (CN); (**b**) Circular beam and forced cooling (CF); (**c**) Square beam and natural cooling (SN); (**d**) Square beam and forced cooling (SF).

**Figure 6:**
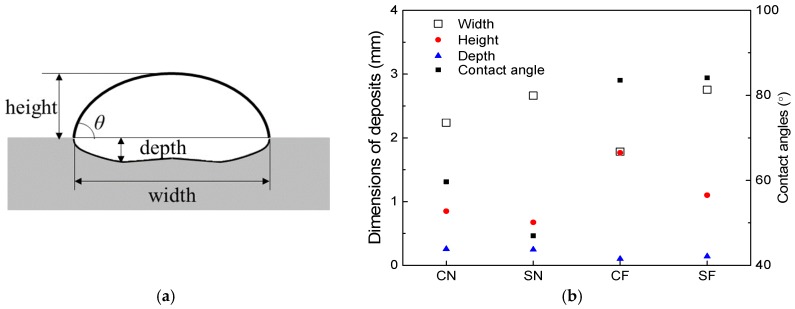
Dimensions of deposits made with four different conditions. (**a**) Definition of geometric parameters; (**b**) Dimensions of deposits for different conditions.

The cross-sections of the deposits fabricated under the four sets of conditions and observed with an OM are shown in Figure 5, and the geometric dimensions of the deposits are measured and plotted in Figure 6.

Comparing Figure 5a with Figure 5c, it can be seen that under the natural cooling condition, a wider and thinner deposit is formed with the square beam spot than that formed with the circular beam spot. The depths of the deposits are basically the same for the two beam profiles. This influence of the beam profiles on the geometric morphology of deposits can also be found under forced cooling conditions by comparing Figure 5b with Figure 5d. 

The forced cooling on the bottom surface of specimens has a significant effect on the morphology of the deposits. From Figure 5a,b, it can be seen that with the circular beam spot, a much thicker (higher) and slightly narrower deposit is formed under the forced cooling condition than that formed under the natural cooling conditions; the depth of the deposit formed with forced cooling is smaller than that formed under natural cooling conditions. From the deposits fabricated using square beam spot shown in Figure 5c,d, it can be seen that a much thicker deposit with smaller depth is also formed under forced cooling conditions than that under natural cooling conditions, but the widths of deposits are comparable under the two cooling conditions. Moreover, the application of the forced cooling results in much larger contact angles between the deposit layers and the substrates, which can be seen from Figure 5 and are also provided in Figure 6. 

### 3.2. Characteristics of Columnar Grains

**Figure 7:**
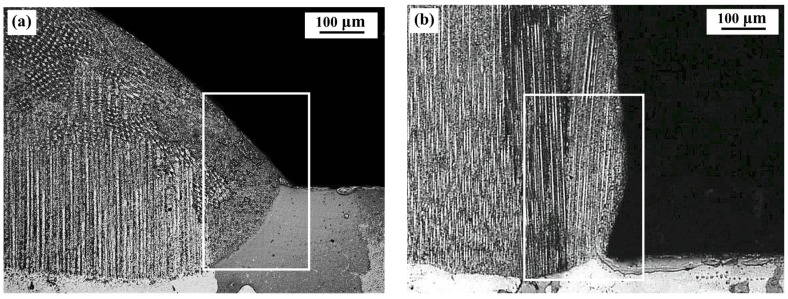

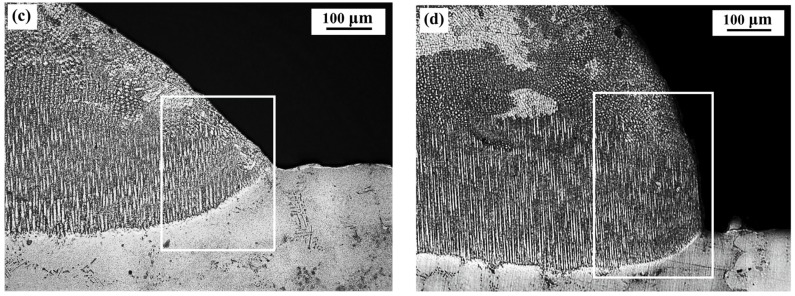
Columnar grains growth at edges of deposits under four different conditions (observed with an OM). **(a**) CN; (**b**) CF; (**c**) SN; (**d**) SF.

**Figure 8:**
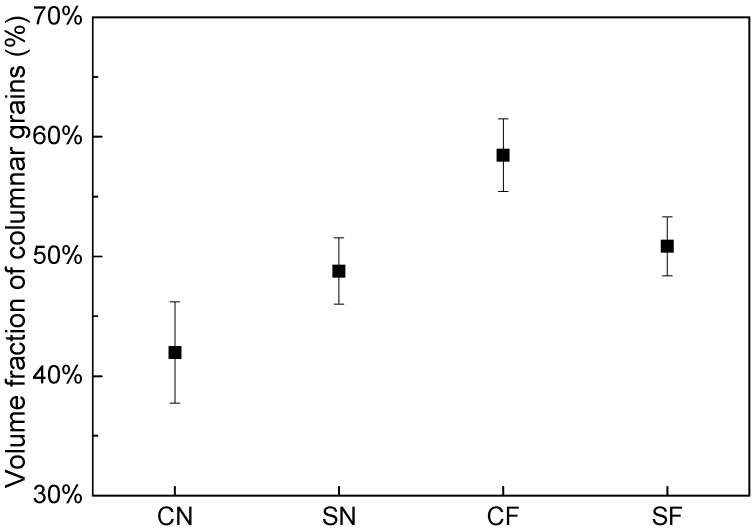
Volume fractions of columnar grains in deposits made under the four conditions.

From Figure 5, it can be seen that all deposits are composed of epitaxial columnar grains at the bottom part and equiaxed grains at the top part under the four deposition conditions. However, the distributions and amounts of the epitaxial columnar grains are different in different laser deposits. For a more detailed analysis of the morphology of the columnar grains, Figure 7 presents the optical images of the microstructures at the edge of the four deposits (shown in Figure 5). Figure 8 presents the volume fractions of the columnar grains in the deposits, which are the ratios of the area of columnar grains to the area of deposits [21].

Under the natural cooling conditions, the distribution of columnar grains in deposits is different for the circular and square laser beam profiles, as shown in Figure 7a,c, respectively. It can be seen that the epitaxial columnar grains are not formed at the edges of the cross-sections of the deposits when using the circular beam spot, while the columnar grains are formed at the edges of the deposits when using the square beam spot. Accordingly, the volume fraction of columnar grains (48%) for the square beam is slightly higher than that (42%) for the circular beam. 

Under the forced cooling conditions, the epitaxial columnar grains can be formed at the edges of deposits regardless of the laser beam profiles used in the deposition, as shown in Figure 7b,d. In addition, compared to the natural cooling, the application of forced cooling can significantly increase the lengths of the columnar grains in deposits. For the circular laser beam, the volume fraction of columnar grains is significantly increased from 42% to 58% by applying the forced cooling, owing to both the longer columnar grains and the epitaxial growth of the columnar grains at the edges of deposits. For the square laser beam, a slight increase of 2% (from 48% to 50%) in the volume fraction of columnar grains is achieved by applying the forced cooling, which is not as significant as that for the circular laser beam. 

## 4. Discussion

### 4.1. Relationships between the Thermal Process and Geometric Morphology of Deposits

**Figure 9:**
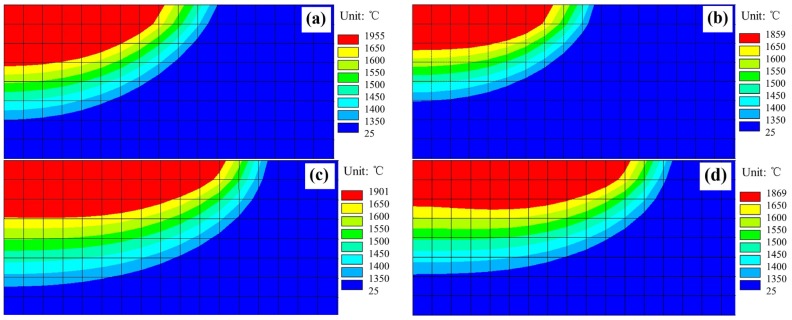
Temperature distributions in cross sections of substrates under the four laser deposition conditions (dimensions of cross-section shown: 1.7 mm × 0.8 mm). (**a**) CN; (**b**) CF; (**c**) SN; (**d**) SF.

With the numerical model developed, the temperature distributions in the cross-sections of the substrates were obtained for the four deposition conditions, as shown in Figure 9. 

It can be seen that no matter which cooling condition is employed, the width of the molten pools are larger for the square laser beam than for the circular laser beam. Under the same cooling conditions, the depths of the molten pools are basically the same for the square and the circular laser beam profiles. The numerical predictions agree well with the experimental findings detailed in Figure 6. 

Obviously, the heat input is lower at the edges than that at center in the width direction when the circular beam profile is used, with a Gaussian distribution of heat flux, as shown in Figure 2a. In contrast, the heat input is uniform and the substrate is heated evenly along the width direction when using the square laser beam, as shown in Figure 2b. This explains why the deposits formed with the square beam are wider than with the circular beam. 

**Figure 10:**
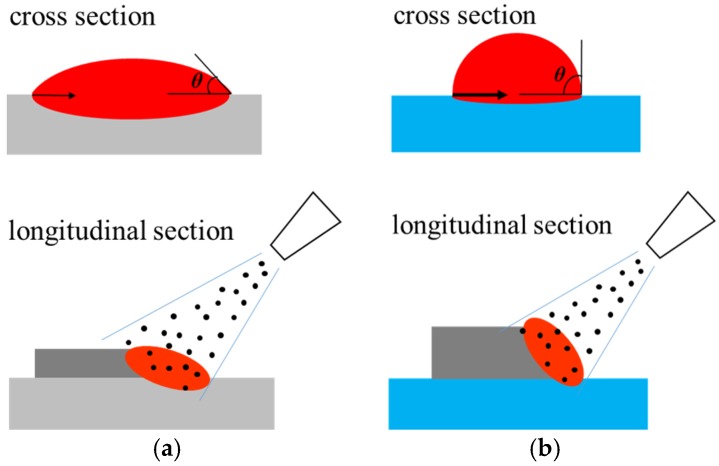
Contact angles and deposit profiles under different cooling conditions. (**a**) natural cooling; (**b**) forced cooling.

It can also be seen from Figure 9 that the depths of the molten pools notably decreased for both the circular and square laser beams when applying the forced cooling condition, which is consistent with the experimental observations. Obviously, the forced cooling can accelerate the heat dissipation from the bottom surface of substrates and reduce the temperature of molten metals more quickly. Such a decrease in temperature will result in the increased surface tension of the molten metal [22] and the spreading of the molten material on the substrate is constrained. Besides, the solidification occurs more rapidly under forced cooling conditions and the spreading time for the molten material on the substrate is shortened. Both the higher surface tension and the shorter time will make the spreading of the molten metal on the substrate surface not as extensive as that under natural cooling conditions. This is why much larger contact angles (given in Figure 6) result from the forced cooling laser deposition, which is schematically shown in Figure 10. Moreover, the larger contact angles can increase the exposure areas of the molten pools to the powders being fed laterally, which allow more powders to enter the molten pools and results in higher deposition layers, as obtained in experiments. A higher deposition rate is therefore possible by employing the forced cooling laser metal deposition. 

### 4.2. Relationships between the Thermal Process and the Growth Direction of Columnar Grains

In existing research [23], it was found that the growth direction of columnar grains is generally opposite to the largest thermal gradient (G) during solidification. Therefore, large thermal gradients in the vertical direction (G_z_) are preferable in the present study for the columnar grains to grow vertically, while the thermal gradients should be smaller in other directions to avoid the formation of stray grains in deposits. 

**Figure 11:**
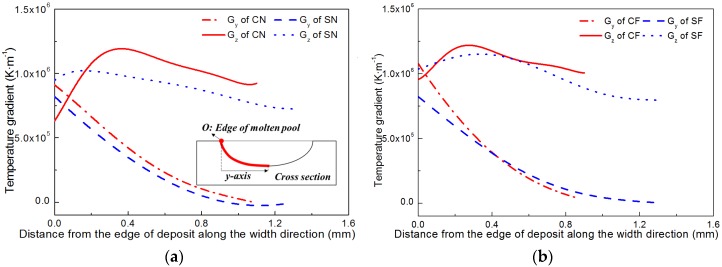
Temperature gradients in front of the solid–liquid interfaces in cross-sections. (**a**) under natural cooling conditions; (**b**) under forced cooling conditions.

The temperature gradients in the width direction (G_y_) and height direction (G_z_) in front of the solid–liquid interfaces are numerically computed on the cross sections of specimens, as shown in Figure 11. It can be seen from Figure 11a that under natural cooling conditions, G_y_ decreases monotonously, while G_z_ first increase and then decrease with the increase of distance from the edge of deposits along the width direction. The G_z_ at the center parts are much larger than G_y_ for both beam profiles, indicating the vertical columnar grains are easier to form in the center than at the edges of a deposit. For the square beam profile, G_z_ is always greater than G_y_, along the whole width of the deposit although the difference between G_z_ and G_y_ is decreasing toward the deposit edge. For the circular beam profile, however, a sharp decrease in G_z_ exists near the deposit edge, resulting in a small region with G_z_ smaller than G_y_, which is favorable for the formation of stray grains. This explains why the columnar grains formed at the deposit edges when using the square laser beam but not when using the circular laser beam, as shown in Figure 7a,c. From Figure 11b it can be seen that by using the forced cooling conditions, G_z_ increased notably at the deposit edges for both beam profiles. For the circular beam profile, the region with G_z_ smaller than G_y_ reduced, which accounts for the observation that the vertical columnar grains can be formed at the edges by applying forced cooling regardless of the beam profiles (shown in Figure 7b,d). 

### 4.3. Relationships between the Thermal Process and the Heights of Columnar Grains

**Figure 12:**
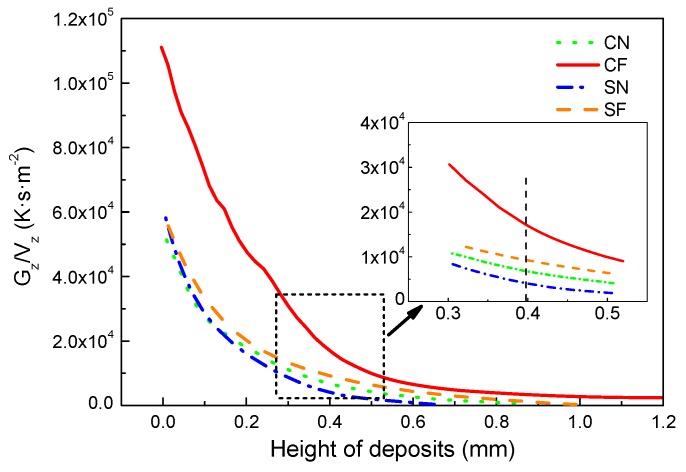
Variation of G_z_/V_z_ with height of deposits at the solid–liquid interface in the central longitudinal section of deposits under four different conditions.

According to the theory of columnar-to-equiaxed transition (CET) during rapid solidification [4,5,7,14], columnar grains will form when G^n^/V is greater than a critical value K, otherwise equiaxed grains will form. Here, G is the temperature gradient, V is the solidification velocity, n and K are positive constants related to materials. Hence, the higher the ratio G/V in front of the solid–liquid interface, the more conducive it is to form the columnar grains. Using the finite element model developed, the temperature gradients in the height direction (G_z_) are calculated at the advancing solid–liquid interface in the central plane (longitudinal section) of deposits under four different conditions. The G_z_/V_z_ obtained are shown in Figure 12. 

It was found that the G_z_/V_z_ decreases with the increase of the height of deposits, indicating a decreasing possibility of the formation of columnar grains, but an increasing possibility of forming equiaxed grains for increased distances from the deposit/substrate interface. This explains why the columnar grains formed on the bottom, while the equiaxed grains formed on the top part of the deposits, as shown in Figure 5. For the same cooling conditions, the G_z_/V_z_ is greater for the circular laser beam than that for the square beam, which agrees with the experimental finding that the columnar grains at the center of deposits formed with the circular laser beam are higher than those with the square laser beam. No matter which laser beam profile is used, the G_z_/V_z_ is greater under forced cooling conditions than that under natural cooling conditions at the same height of the deposits, which indicates an improved condition for the columnar grains to grow under the forced cooling conditions. From the inset in Figure 12, it can be seen that the G_z_/V_z_ has the relationship of CF > SF > CN > SN at the height of 0.4 mm in the four deposits. This means that at the center plane of a deposit, the circular laser beam combined with the forced cooling conditions are the most favorable for the epitaxial growth of columnar grains, while the square laser beam with natural cooling are the worst conditions for the development of columnar grains, which agrees very well with the results shown in Figure 5.

## 5. Conclusions

In this paper, laser deposits were made on a directionally solidified super IC 10 with two types of laser beam profile (circular and square) and two different cooling conditions (natural cooling and forced cooling); the geometric morphology and microstructure of the deposits obtained under different conditions were examined; and the thermal processes during the laser deposition were numerically analyzed. Based on above work, the following conclusions were drawn:

(1) Wider and thinner deposits were obtained with the square laser beam than those with the circular laser beam, no matter whether natural or forced cooling conditions were used. The heights and contact angles of the deposits were notably increased by the application of forced cooling.
(2) Under the natural cooling conditions, the epitaxial columnar grains formed at the edge of the deposits with the square laser beam, but not with the circular laser beam; a higher volume fraction of columnar grains was therefore obtained by using the square beam profile.
(3) Under forced cooling conditions, columnar grains formed at both the center and the edges of the deposits, regardless the laser beam profiles. The volume fractions of columnar grains were increased by applying forced cooling, and the increase was more significant for the circular beam than for the square beam.
(4) Low substrate temperatures and short solidification times of molten metals under forced cooling are beneficial to forming deposits with increased heights and contact angles between deposits and substrates.
(5) High thermal gradients and large ratios of G/V in the height direction can be obtained by adjusting the laser beam profiles and cooling conditions, which are preferable for forming deposits with higher volume fractions of directionally-grown columnar grains.

## Figures and Tables

**Table 1:** Chemical composition of IC10 alloy (wt %).

Elements	C	Co.	Cr	Al	W	Mo	Ta	Hf	B	Ni
Content, wt %	0.1	12.0	7.0	5.9	5.0	1.5	7.0	1.5	0.015	Balance

**Table 2:** Four sets of experiments carried out in the study.

Conditions	Forced Cooling (F)	Natural Cooling (N)
Circular laser beam spot with a Gaussian distribution of power density (C)	Circular beam with forced cooling (CF)	Circular beam with natural cooling (CN)
Square laser beam spot with an uniform power density in one direction and a Gaussian distribution in another (S)	Square beam with forced cooling (SF)	Square beam with natural cooling (SN)

**Table 3:** Thermo-physical property parameters of IC10 used in numerical modeling.

Property	Values
Density (kg/m^3^)	8290
Specific heat (J/kg·°C)	360 + 0.04 × T (°C)
Thermal conductivity (W/m·°C)	8.43 + 0.0178 × T (°C)
Solidus temperature (°C)	1337
Liquidus temperature (°C)	1370
Latent heat of fusion (J/kg)	3.0 × 10^5^
